# MD Simulation Reveals a Trimerization-Enhanced Interaction of CD137L with CD137

**DOI:** 10.3390/ijms26051903

**Published:** 2025-02-22

**Authors:** Hefeng Wang, Jianhua Wu, Ying Fang, Quhuan Li

**Affiliations:** School of Biology and Biological Engineering, South China University of Technology, Guangzhou 510006, China; 201910108090@mail.scut.edu.cn (H.W.); wujianhua@scut.edu.cn (J.W.)

**Keywords:** TNF, immunotherapy, CD137L, CD137, monomer, dimer, trimer, MD simulation, binding affinity, structural stability

## Abstract

CD137 is a prominent costimulatory molecule of the tumor necrosis factor (TNF) receptor superfamily that activates T cells through a complex bidirectional signaling process involving CD137L. The clinical value of immunotherapies underscores the potential of CD137L/CD137 as an effective target for boosting antitumor immune responses; however, the intricate mechanisms governing these interactions have not been fully elucidated. Herein, we constructed various oligomeric states of CD137L (monomeric, dimeric, and trimeric CD137L) and explored their interactions with CD137 using molecular dynamics simulations. Our findings revealed that trimeric CD137L exhibits higher thermal stability but reduced binding affinity for CD137 compared with the dimer form, with the A’B’ loop of CD137L playing a critical role in both structural stability and promoting CD137 interactions. Notably, the formation of hexameric structures enhanced the binding affinity and stability. This study provides valuable insights into the CD137L/CD137 bidirectional signaling mechanisms, which may inform the design of next-generation CD137 agonists. Ultimately, these advancements may improve cancer immunotherapy strategies, aiming to enhance therapeutic outcomes for patients through more effective and targeted therapies.

## 1. Introduction

The tumor necrosis factor superfamily (TNFSF) and its receptors (TNFRSF) are crucial regulators of the immune system [1,2,3]. Interactions between TNFRSF and their corresponding ligands are essential for T-cell activation and the development of T-cell immunity [4]. Additionally, these interactions activate ligand-dependent signaling pathways, often referred to as “reverse signals”, that trigger cellular responses [5,6,7]. These signaling pathways are integral to the regulation of various aspects of immune cell biology, including survival, proliferation, inflammation, apoptosis, and lymphocyte homeostasis [8,9]. CD137, also known as 4-1BB and TNFRSF9, is a notable costimulatory receptor of the TNFRSF family. Over the past 20 years, it has emerged as a promising therapeutic target for enhancing antitumor immune responses in both preclinical and clinical settings [10]. The interaction between CD137 and its ligand, CD137L (4-1BBL or TNFSF9), triggers bidirectional signals that are vital for maintaining immune equilibrium [11,12].

CD137 is predominantly expressed on activated T and natural killer (NK) cells [13], whereas CD137L is primarily present in activated antigen-presenting cells, such as activated B cells, macrophages, and dendritic cells [14]. When CD137 binds to CD137L, it recruits TNFR-associated factor (TRAF) 1 and TRAF2, activating signals through the pivotal transcription factor NF-κB and mitogen-activated protein kinases (MAPKs) [15,16]. This coactivation leads to increased proliferation and survival of CD8^+^ T and NK cells, along with the increased secretion of proinflammatory cytokines, enhanced cytolytic activity, and improved antibody-dependent cell-mediated cytotoxicity [9,17,18]. CD137L engagement activates protein tyrosine kinases, p38 mitogen-activated protein kinase (MAPK), extracellular signal-regulated kinase (ERK), MAPK/ERK (MEK), phosphoinositide-3-kinase (PI3K), and protein kinase A (PKA) in human monocytes [17]. Namely, the inflammation mediated by Toll-like receptor 4 relies on CD137L to maintain tumor necrosis factor (TNF)-α production during late-phase signaling [19]. Moreover, when CD137L signals into monocytes, it drives their differentiation into CD137L-dendritic cells, which preferentially promote the polarization of type 1 T helper (Th1) cells and robust type 1 CD8^+^ T cell responses against tumor-associated viral antigens [14]. Reverse CD137L signaling induces the differentiation of monocytes to CD137L-DCs. CD137L-DCs induce potent Th1 and type 1 CD8^+^ T cell (Tc1) responses, leading to strong immune responses against virus-associated tumor cells, making them a promising candidate for virus-associated cancer immunotherapy [14]. Thus, the interaction between CD137 and CD137L initiates complex signaling cascades that are essential for the immune response to malignancies [20].

CD137 signaling plays a vital role in T cell activation and has emerged as a promising target for cancer immunotherapy [21,22,23]. However, the development of CD137 agonists in clinical settings has encountered significant hurdles, particularly in achieving an optimal balance between efficacy and systemic toxicity [24,25]. For instance, the IgG4 antibody urelumab (BMS-663513) was discontinued due to severe-to-fatal liver toxicity, which affected over 5% of patients, despite its effectiveness against melanoma [26,27]. Although the trials were resumed in 2012, the combination of urelumab with cetuximab or nivolumab in patients with solid tumors did not demonstrate a clear additive benefit in terms of preliminary response rates [28]. Utomilumab (PF-05082566) showed improved safety with fewer severe adverse effects and no dose-limiting toxicity, but with low clinical efficacy, benefiting only a limited number of patients with immune-responsive cancers [29,30]. A novel approach is represented by STA551, which selectively activates CD137 in the presence of ATP, targeting the tumor microenvironment while minimizing systemic toxicity [31]. Additionally, ADG106, a human anti-CD137 IgG4 agonist antibody, binds to the junction of CRD2 and CRD3, overlapping the CD137L binding site within these domains [32]. The conformation of CD137 when associated with various agonist antibodies, such as urelumab, utomilumab, and ADG106, displays a pattern that reflects their functional activities in relation to CD137L [24]. JNU-0921, another agonist, has been shown to effectively reduce tumor burden by enhancing the cytotoxicity of CD8^+^ T cells in cis and in trans [33]. These studies aim to leverage the differences between tumor and normal tissues to achieve preferential tumor targeting and mediate CD137 crosslinking, thereby achieving potent activation while reducing systemic toxicity and maintaining antitumor efficacy [24]. CD137 agonists rely on receptor clustering to achieve effective signaling; however, current antibody designs exhibit significant mechanistic limitations. Some agonists, like utomilumab, induce only limited receptor clustering, resulting in suboptimal activation, whereas others, such as urelumab, cause systemic toxicity due to excessive activation [30]. Additionally, many agonists depend on FcγRIIb-mediated crosslinking, limiting their efficacy in FcγRIIb-deficient immune environments [24]. Given these challenges, a deeper understanding of CD137L-CD137 interactions is essential for designing next-generation agonists that achieve optimal receptor clustering without relying on FcγRIIb engagement.

The homotrimeric structure of TNFSF represents its biologically active form; however, this form is unstable in the physiological environment [34,35]. Non-covalent interactions maintain monomeric CD137L as a compact, bell-shaped trimer [30,36,37]. Of note, the mechanisms underlying the trimerization of CD137L and CD137L/CD137 interactions, which are required to activate the above described intracellular signaling pathways [38,39,40], remain to be clarified. This study aimed to address this knowledge gap by constructing and analyzing different oligomeric states of CD137L (monomeric, dimeric, and trimeric CD137L) and investigate their interactions with CD137. Using molecular dynamics (MD) simulations, our findings suggest that triCD137L has a reduced binding affinity for CD137 compared with diCD137L, exhibiting higher thermal stability. The A’B’ loop of CD137L is an important regulator of trimer stability and its interaction with CD137. Furthermore, the binding affinity and stability were further enhanced in the formation of hexameric CD137L/CD137 complex, which may pave the way for innovative strategies for drug design. This detailed knowledge on the molecular features of CD137L/CD137 interactions may provide new foundations for the development of more effective CD137-based immunotherapies.

## 2. Results

### 2.1. CD137L Trimer Has Better Thermostability than the Dimer Complex

**Figure 1:**
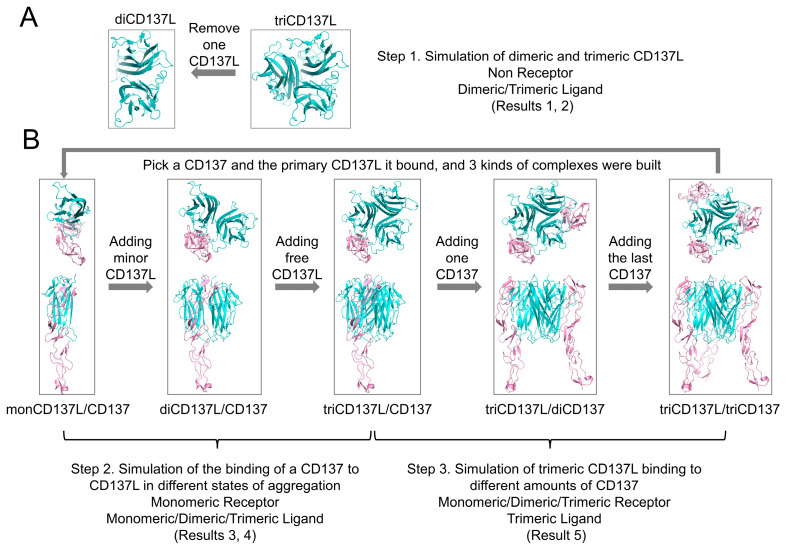
System construction. (**A**) CD137L trimer (PDB ID: 6MGE) was the initial crystal structure. One of the three monomer CD137L (monoCD137L) units was removed to create three CD137L dimer (diCD137L) complexes. (**B**) The crystal structure of the CD137L trimer bound to three CD137 molecules (triCD137L/triCD137) is available in the Protein Data Bank (PDB ID: 6A3V). We selected one CD137 along with its primary binding partner CD137L to construct three CD137L/CD137 complexes. Next, an additional CD137L was introduced (referred to as the minor CD137L) to each CD137L/CD137 complex, resulting in three complexes of diCD137L/CD137. Subsequently, the final CD137L, which does not bind to CD137, was added (designated as free CD137L). This allowed us to form three complexes of triCD137L bound to CD137 (designated as triCD137L/CD137). To simulate the binding process of triCD137L with one, two, and three CD137, ultimately forming a hexamer, a second CD137 was added to the triCD137L/CD137 system, generating three complexes of triCD137L bound to two CD137 (denoted as triCD137L/diCD137). Finally, the hexamer comprising triCD137L bound to three CD137 was designated of triCD137L/triCD137. CD137L is represented in blue, while CD137 is shown in pink.

**Figure 2:**
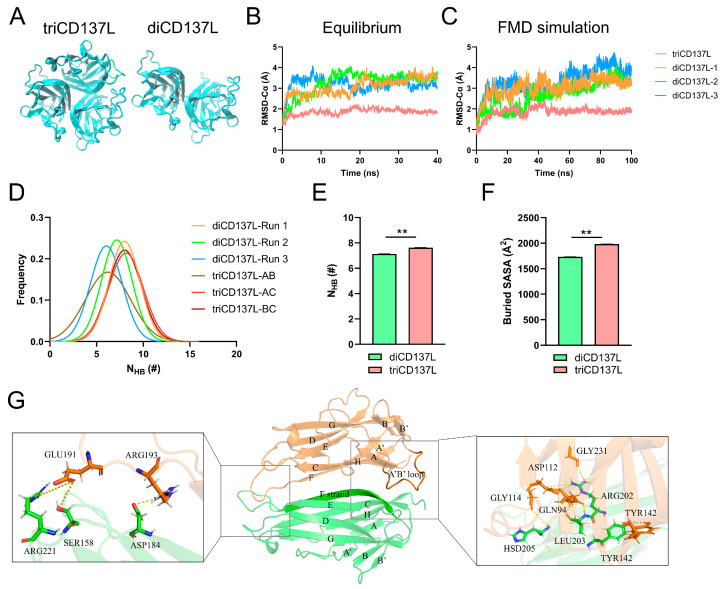
Structure and stability of triCD137L and diCD137L. (**A**) Crystal structure of triCD137L (left) and one representative diCD137L (right) are represented. (**B**) Time course of Cα root-mean-square deviation (RMSD) of triCD137L and three dimeric CD137L complexes during a 40 ns equilibrium. (**C**) One stable conformation was selected from each of the equilibrium complexes and a 100 ns free molecular dynamics (FMD) simulation was performed. (**D**) Distribution frequency, (**E**) number of hydrogen bonds, and (**F**) buried SASA between monomers in triCD137L and diCD137L were analyzed over a 100 ns FMD simulation. (**G**) Binding sites of two interacting monomers (in orange and green, respectively) were revealed by FMD. The uppercase letters (middle inset) represent different regions of CD137L, with each letter corresponding to a specific strand. The crucial binding subsite (right inset) was located in sheets, whereas another subsite (left inset) was in loops. All data are shown as mean ± standard error of the mean (SEM) (*n* = 3), and ** indicates *p* < 0.001.

First, we constructed systems of diCD137L and triCD137L, which were composed of three and two CD137L monomers (Figure 1A and Figure 2A), respectively, to investigate the structural stability of dimeric and trimeric CD137L. To obtain stable conformations of triCD137L and diCD137L complexes, we performed 40 ns equilibrium MD simulations for each system. The time course of Cα atom root-mean-square deviation (RMSD) indicated that the four complexes were in an equilibrium state, as evidenced by their stable plateau positions (Figure 2B). After reaching equilibrium, we conducted a 100 ns free molecular dynamics (FMD) simulation of each complex. The Cα-RMSD of triCD137L stabilized at approximately 2 Å after the initial 10 ns, whereas that of diCD137L fluctuated between 3 and 4 Å, and remained within 1 Å during the final 50 ns (Figure 2C). The Gaussian fit of the hydrogen bond distribution suggests that the simulation duration was adequate to capture all relevant conformational changes and stability dynamics (Supplementary Figure S1). These results indicated that both triCD137L and diCD137L were stable, and that the monomers bound tightly.

Next, we calculated the distribution frequencies of hydrogen bonds (H-bonds) between the two adjacent monomers of triCD137L and diCD137L. The distribution frequencies of the number of H-bonds (N_HB_) were fitted to Gaussian distributions (Figure 2D). We also measured the number of H-bonds and the solvent-inaccessible surface area (buried SASA) between the two interacting monomers in triCD137L and diCD137L (Figure 2E,F). The average interfacial H-bonds and buried SASA of diCD137L were 7.13 and 1735.34 Å^2^, respectively, and were higher in triCD137L (7.62 and 1982.67 Å^2^), suggesting that triCD137L had better thermostability than the dimer form. We also investigated the H-bonds formed between the residue pairs within the binding site of the two monomers during a 100 ns FMD simulation. H-bonds with occupancies greater than 10% were observed in both triCD137L and diCD137L complexes. One binding subsite (located on the right) was found between beta sheets and loops, whereas another subsite (situated at the left) was present within loops (Figure 2G). The residues ARG202 and HSD205 of the F strand in one monomer were found to establish H-bonds with residues ASP112 and GLY114 of the A’B’ loop in another monomer within the appropriate binding subsite. Residue LEU203 in the F strand also interacted with GLN94. In addition, H-bonds were formed between the TYR142 residues of the two monomers. The H-bonds formed by residues GLU191 and ARG193 in the loop, which linked strands E and F to residues ARG221, SER158, and ASP184, constituted the bottom-binding subsite. These residues are important for dimerization and trimerization.

### 2.2. Key Residues and Structural Variations Are Responsible for Improved triCD137L Thermostability

**Figure 3:**
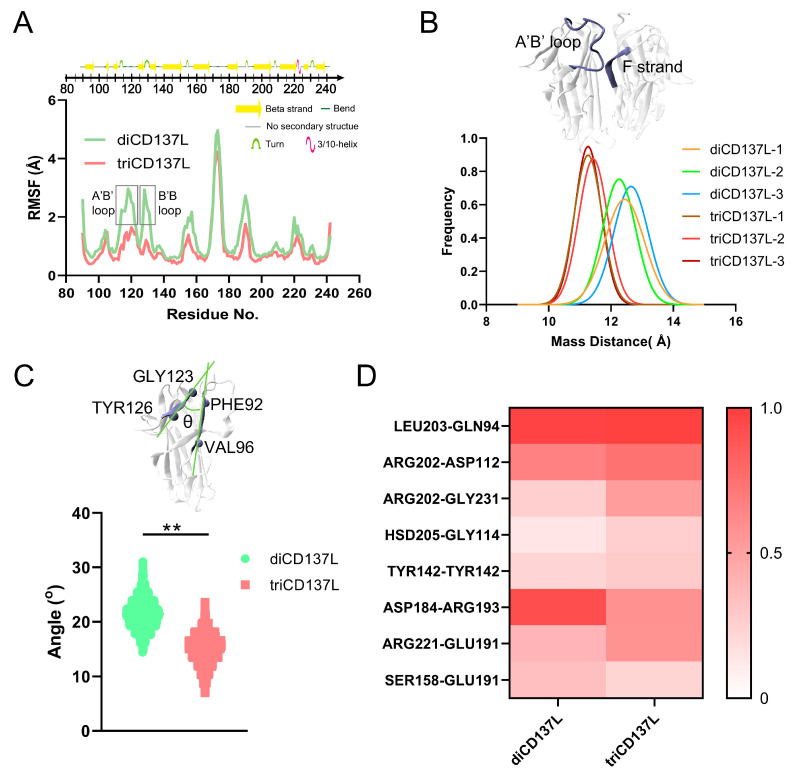
Structural basis the higher stability of triCD137L. (**A**) Cα-RMSF patterns of residues in diCD137L and triCD137L. (**B**) Mass distance between A’B’ loop (residues ASP112–GLY123) in one monoCD137L and residues GLN200–LEU206 of the F strand in the adjacent monoCD137L. (**C**) Angle θ was quantified by the cross angle between residues GLY123 and TYR126 in the B’ strand and the PHE92 and VAL96 residues in the A strand. (**D**) Heatmap showing the occupancy of H-bonds between residue pairs involved in binding two adjacent monoCD137L in diCD137L and triCD137L during the 100 ns FMD simulations. All data are shown as mean ± SEM, (*n* = 3), and ** indicates *p* < 0.001.

To characterize the structural variations responsible for the improved thermostability of triCD137L, we compared the Cα RMSF values of residues between triCD137L and diCD137L. The results revealed significant variation in the A’B’ loop (residues ASP112–GLY123) and B’B loop (residues GLU128–LYS131) (Figure 3A). The A’B’ and B’B’ loops are highly conserved among primates (Supplementary Figure S2), as analyzed by the ENDscript server [41]. Thus, their structural stability may be crucial for CD137L-mediated receptor binding and immune activation. Given that the key residues ASP112 and GLY114 are located in the A’B’ loop, where they establish H-bonds with ARG202 and HSD205 in the adjacent monomer, we determined the distribution frequencies of mass distance (DMC) between the A’B’ loop in one monomer and residues GLN200–LEU206 of the F strand in the neighboring monomer. During the 100 ns FMD simulation, the DMC in the triCD137L was mainly distributed between 10 and 12 Å, but in diCD137L, it was mainly distributed between 12 and 14 Å (Figure 3B). The B’ and B strands were linked by the B’B loop, and these secondary structures exhibited significant fluctuations in diCD137L. To quantify these fluctuations, we measured the cross angle between residues GLY123 and TYR126 in the B’ strand with residues PHE92 and VAL96 in the A strand, which exhibited stability throughout a 100 ns FMD simulation. However, this angle significantly increased in diCD137L (Figure 3C). These results indicate that the proximity of the A’B’ loop to the F strand favors binding between the two monomers and that the up-and-down oscillation of the B strand destabilizes the complex.

To further elucidate the structural basis for the enhanced thermostability of triCD137L, we examined next the crucial H-bonds formed between the residue pairs at the binding site during 100 ns FMD simulations. We identified eight H-bonds across the interface of two adjacent monomers, with occupancy exceeding 10% in both the diCD137L and triCD137L systems (Figure 3D). Notably, the occupancy of key H-bonds, including ARG202–ASP112, ARG202–GLY231, and HSD205–GLY114, was significantly increased in triCD137L. The residues ARG202 and HSD205 are located in the F strand, while ASP112 and GLY114 are in the A’B’ loop. The closer proximity of the F strand to the A’B’ loop in triCD137L enhances interaction opportunities between CD137L monomers, thereby stabilizing the trimeric assembly and contributing to its increased thermostability. Additionally, the occupancy of other H-bonds, including ARG221–GLU191, and TYR142–TYR142, was also significantly increased in triCD137L, highlighting their importance in trimerization. However, other H-bonds, such as ASP184–ARG193 and SER158–GLU191, were decreased in triCD137L, thus primarily contributing to the stability of diCD137L.

### 2.3. CD137L Dimerization and Trimerization Enhances Its Binding Affinity to CD137

**Figure 4:**
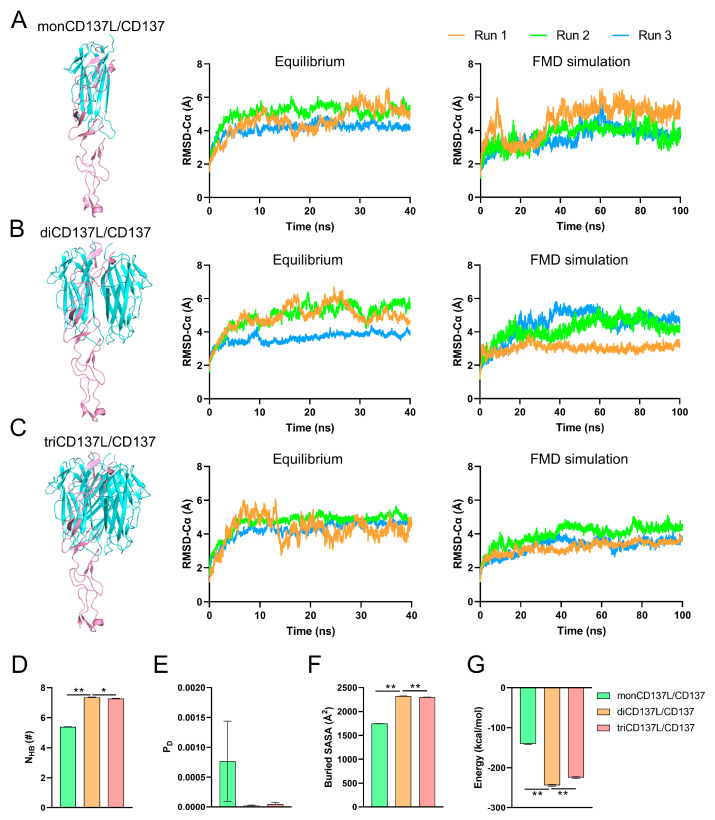
Dimerization and trimerization of CD137L are beneficial for binding to CD137. Front view and Cα-RMSD of (**A**) monCD137L/CD137, (**B**) diCD137L/CD137, and (**C**) triCD137L/CD137 complex systems. CD137L is represented in blue, while CD137 is shown in pink. Each complex system encompassed three distinct combinations, as outlined in Supplementary Table S1. Cα-RMSD time course was calculated for the system lacking the CRD4 domain of CD137. (**D**) Number of H-bonds (N_HB_), (**E**) dissociation probability (P_D_), (**F**) buried solvent-accessible surface area (buried SASA), and (**G**) energy between CD137 and all its bound CD137L. * *p* < 0.05 and ** *p* < 0.001 as determined by Tukey’s multiple comparisons test. All data are shown as mean ± SEM (*n* = 3).

As a member of the TNFSF, CD137 conventionally binds to CD137L, forming a complex where one CD137 molecule interacts with two CD137L molecules. Interestingly, in contrast to most TNF family members, CD137 preferentially binds to a specific CD137L molecule and displays only minor interactions with others [37]. As 92% of the buried surface area is contributed by the interaction with primary CD137L, this uneven binding may suggest that the interaction does not require the formation of a complete ligand trimer [37]. To investigate the effect of CD137L polymerization on CD137 binding, we established three distinct systems: monoCD137 bound to monoCD137L (CD137L/CD137), diCD137L (diCD137L/CD137), and triCD137L (triCD137L/CD137) (Figure 1B and Figure 4A). For each system, we conducted a 40 ns equilibrium simulation (Figure 4B) followed by a 100 ns FMD simulation (Figure 4C). Each CD137L in triCD137L exhibited a distinct binding interface with monoCD137, categorized as primary, minor, or free CD137L, based on the size of their respective binding surfaces. The Cα atom RMSD of the protein in all complexes fluctuated widely during the 40 ns equilibrium, but CD137L remained stable with a Cα atom RMSD of approximately 3 Å (Supplementary Figure S3).

The CRD4 domain (one of the CD137 domains) is connected to the CRD1–3 domains of CD137 through a loop and is located distantly from the binding interface of the complexes. Consequently, this domain exhibits unrestricted motion and significant fluctuations. This explains the wide fluctuation in the observed RMSD values for the complexes compared with those of CD137 without the CRD4 domain (Supplementary Figure S3). To investigate the equilibrium status of these systems, we computed the RMSD of Cα atoms in the entire complexes excluding CRD4. Our results demonstrated that all three complexes were in an equilibrium state, as evidenced by their stable plateau-like RMSD values during the 40 ns equilibration period, indicating the stability of the binding interface (Figure 4B). Furthermore, we also computed the RMSD of the Cα atoms for the entire complexes, excluding CRD4, during a 100 ns FMD simulation (Figure 4C). These analyses revealed that these complexes remained stable throughout the simulation period. To elucidate the impact of CD137L aggregation on CD137 binding, we assessed various parameters, including NHB, dissociation probability (PD), buried SASA, and binding energy between all bound CD137L and CD137 molecules. The higher NHB and buried SASA, and lower PD and energy in the diCD137L/CD137 and triCD137L/CD137 complexes suggested that minor CD137L contributed to the binding of CD137 (Figure 4D–G). Notably, the binding affinity between CD137 and CD137L in diCD137L/CD137 was significantly greater than that in triCD137L/CD137. These results suggest that the dimerization and trimerization of CD137L augment its binding affinity toward CD137, with diCD137L exhibiting the highest binding affinity. Our findings suggest that the impact of ligand oligomerization on binding affinity and stability observed in CD137L/CD137 can be generalized to other TNF superfamily ligands and receptors, particularly those with symmetric receptor-ligand interactions.

**Figure 5:**
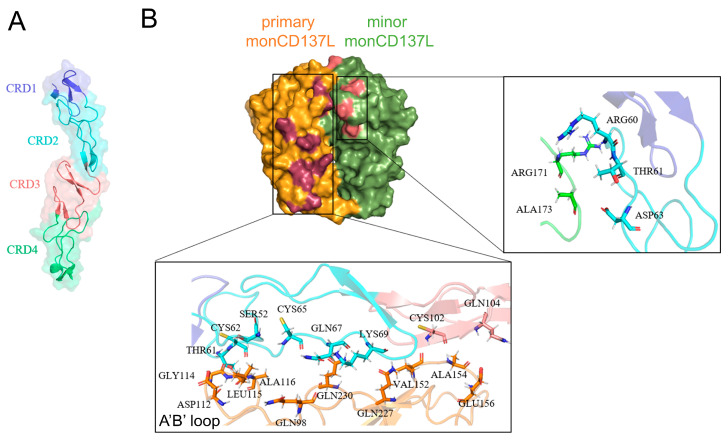
Binding sites of CD137 with primary and minor CD137L. (**A**) Schematic diagram of CD137 domains. (**B**) Overview of the binding sites of CD137 with primary (orange) and minor (green) CD137L molecules. Interactions between primary monCD137L and CD137 are shown in deep red while interactions between minor monCD137L and CD137 are shown in pink. Bottom and right insets show the CD137 binding sites with primary and minor CD137L, respectively. Key residues involved were depicted.

To elucidate the binding structure formed between CD137 and the two monomeric forms of CD137L, we visualized the specific binding sites of these ligands on CD137 (Figure 5). The extracellular region of CD137 comprised four domains: CRD1, CRD2, CRD3, and CRD4 (Figure 5A); among which, CRD2 and CRD3 are known to play pivotal roles in mediating interactions with CD137L. The primary binding site was identified where the A’B’ loop within monoCD137L actively engaged with CD137 (Figure 5B, low inset). Additionally, ARG171 and ALA173 residues within the minor monoCD137L formed H-bonds with ARG60, THR61, and ASP63 residues in CD137 to establish a secondary binding site (Figure 5B, right inset).

### 2.4. Allosteric Modulation of CD137 Binding Affinity Through Conformational Dynamics and Hydrogen Bonding in CD137L Complexes

**Figure 6:**
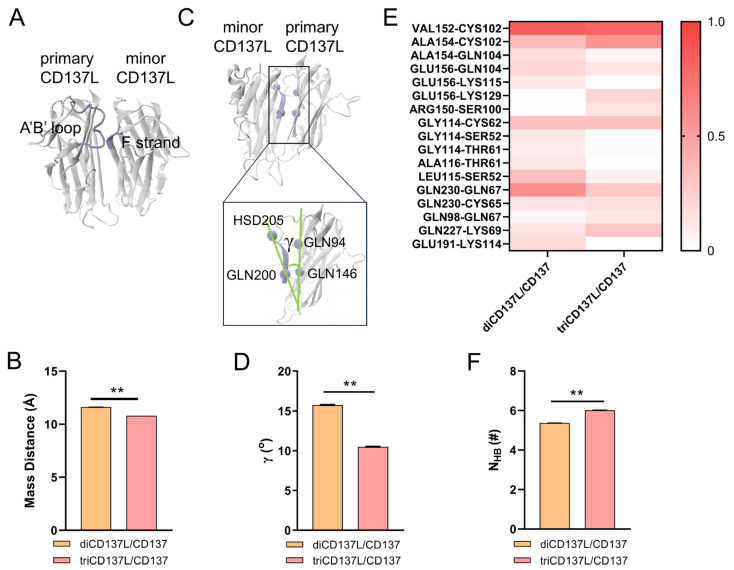
Allosteric regulation of the A’B’ loop in controlling the CD137L/CD137 binding affinity and CD137L trimerization. (**A**,**B**) Mass distance between A’B’ loop on the primary CD137L and F strand (residues from ARG202 to HSD205) in the minor CD137L was measured over 100 ns FMD. (**C**,**D**) Angle γ between the primary and minor CD137Ls was estimated by the cross angle of two lines connecting the Cα atoms of GLN200 and HSD205 residues in the minor CD137L, as well as GLN146 and GLN94 residues in the primary CD137L, over 100 ns FMD. (**E**) Heatmap showing H-bonds occupancy between residue pairs involved in the interaction of primary CD137L with CD137 during 100 ns FMD. (**F**) Number of H-bonds between the primary and minor CD137Ls during 100 ns FMD. All data are shown as mean ± SEM (*n* = 3), and ** indicates *p* < 0.001.

To uncover the allosteric regulation that governs the higher binding affinity between CD137L and CD137 in diCD137L/CD137, we analyzed conformational changes using a 100 ns FMD simulation. We measured the mass distance between the A’B’ loop in the primary CD137L and the residues ARG202 to HSD205 of the F strand in minor CD137L. Compared with diCD137L/CD137, the mass distance in triCD137L/CD137 was reduced, which indicates that the A’B’ loop in triCD137L/CD137 is closer to the F strand and farther away from CD137 (Figure 6A,B). However, the A’B’ loop was found to play a crucial role in the binding of primary CD137L to CD137, indicating that a longer distance between the A’B’ loop and CD137 is unfavorable for this interaction. The proximity of the A’B’ loop in primary CD137L to CD137 was increased in diCD137L/CD137 compared with that in triCD137L/CD137, facilitating stronger interactions and resulting in higher binding affinity.

The angle γ formed by the line connecting residues GLN94 and GLN146 in the primary CD137L and the line connecting the GLN200 and HSD205 residues of the F strand in the minor CD137L was further measured (Figure 6C,D). Of note, the angle was smaller in triCD137L/CD137 complexes, suggesting a closer proximity between the primary and minor CD137Ls. Although this proximity enhances the triCD137L structural stability, it may compromise its binding to CD137. To verify this hypothesis, we compared the occupancy of key H-bonds between the primary CD137L and CD137 in the diCD137L/CD137 and triCD137L/CD137 systems (Figure 6E). Specifically, the H-bond occupancy in the A’B’ loop between the primary CD137L and CD137, such as GLY114–CYS62, LEU115–SER52, and ALA116–THR61, was higher in the diCD137L/CD137 complex. Furthermore, we also calculated the N_HB_ between the primary and minor CD137Ls in the diCD137L/CD137 and triCD137L/CD137 complexes. More H-bonds were observed in the triCD137L/CD137 system (Figure 6F), which is consistent with our previous results (Figure 2E). These data show that primary and minor CD137Ls bind more closely in the triCD137L/CD137 complexes and that the A’B’ loop is important for both receptor binding and ligand trimerization. Thus, diCD137L is more favorable for binding because it minimizes steric constraints, allowing the A’B’ loop to form stronger and more stable interactions with CD137. These results suggest that this increased stability, arising from enhanced interactions between the A’B’ loop of the primary CD137L and the F strand of the minor CD137L, may compromise functional flexibility.

### 2.5. Trimerization of monoCD137 Induced by triCD137L Enhances the Binding Affinity Between CD137L and CD137 and CD137L Stability

**Figure 7:**
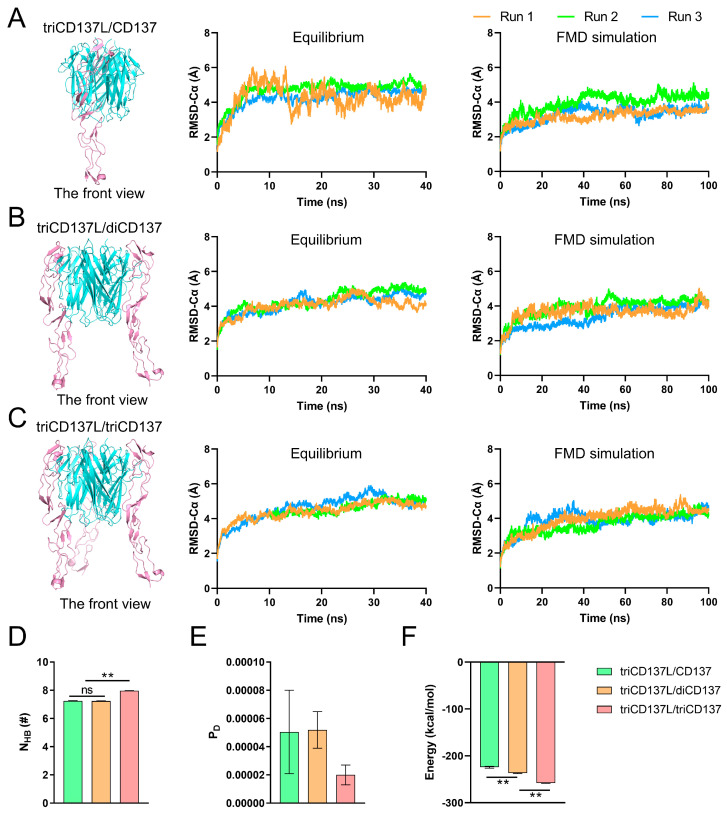
Binding affinity was increased as more triCD137L bound to CD137. Front view and Cα atom RMSD of the (**A**) triCD137L/CD137, (**B**) triCD137L/diCD137, and (**C**) triCD137L/triCD137 complex systems. Each complexes underwent 40 ns equilibrium and 100 ns FMD simulations, thrice. (**D**) Number of H-bonds, (**E**) dissociation probability P_D_, and (**F**) energy between triCD137L and CD137/diCD137/triCD137 were calculated during a 100 ns FMD, thrice. All data are presented as the mean ± SEM (n ≥ 3), and ** indicates *p* < 0.001.

CD137L trimerization promotes the aggregation of three CD137 receptors, which exists as a monomer in T cells [42]. This mechanistic process triggers specific signaling pathways that will directly impact on the activation, proliferation, and enhanced survival of cells [20]. To simulate this process, we established three types of systems: triCD137L bound to one (triCD137L/CD137), two (triCD137L/diCD137), and three (triCD137L/triCD137) CD137s (Figure 1B and Figure 7A–C). Subsequently, we conducted a 40 ns equilibrium simulation followed by a 100 ns FMD simulation for each system, repeated thrice.

The Cα atom RMSD of the entire complexes, excluding the CRD4 domain, was calculated during 40 ns equilibrium and 100 ns FMD. We observed that these systems were all in a stable state, either during equilibrium or FMD, as the Cα atom RMSD of the complexes remained around 4 Å after the first 10 ns (Figure 7A–C). To investigate the impact of triCD137L-induced trimerization on the binding affinity between CD137L and CD137, we calculated the N_HB_, P_D_, and interaction energy between CD137 molecules and all bound CD137L molecules, including primary and minor CD137Ls. N_HB_ was significantly increased in triCD137L/triCD137 systems, whereas P_D_ was reduced (Figure 7D,E). Moreover, the binding energy decreased as more CD137 bound (Figure 7F). These results suggest that an increasing number of bound CD137 molecules enhance the binding affinity and stability of the CD137L/CD137 interaction during the evolutionary process of trimeric CD137L-induced trimerization of CD137.

**Figure 8:**
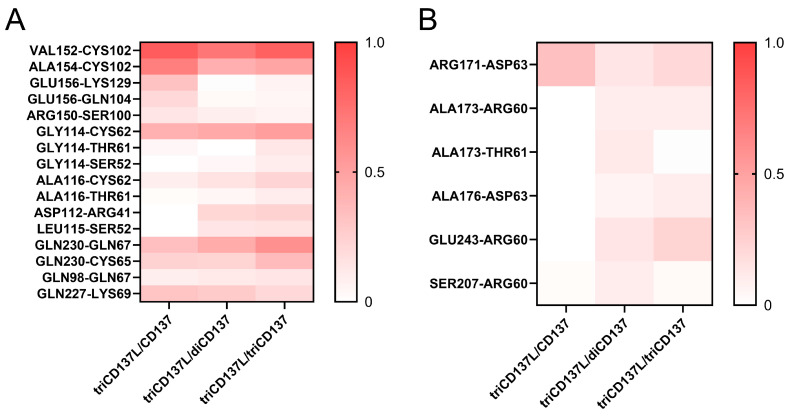
Heatmap of H-bond occupancies of residue pairs involved in the interaction between CD137L and CD137 during 100 ns FMD simulation. (**A**) Residue pairs involved in the primary CD137L/CD137 interaction. (**B**) Residue pairs involved in the minor CD137L/CD137 interaction.

To further elucidate the structural basis underlying the enhanced binding affinity between CD137L and CD137, we investigated the H-bond interactions between residue pairs at the binding site during FMD simulation (Figure 8). The occupancy of H-bonds, such as GLY114–CYS62, GLY114–THR61, GLY114–SER52, ALA116–CYS62, ALA116–THR61, ASP112–ARG41, LEU115–SER52, and GLN230–GLN67 in primary CD137L increased with increasing CD137 binding (Figure 8A). These data confirmed that A’B’ loop was also important in CD137L-induced CD137 trimerization. Although the H-bonds involved in the binding of minor CD137L to CD137 were transient, their occupancy regarding ALA176–ASP63 and GLU243–ARG60 increased with increasing number of bound CD137 molecules (Figure 8B). The minor CD137L forms only one or two hydrogen bonds with CD137, and these interactions were intermittent, suggesting that while it plays a role in CD137 engagement, its direct contribution to receptor binding is limited. Although the minor CD137L is not a major determinant of CD137 binding affinity, its presence may help maintain the structural arrangement necessary for optimal receptor engagement and signal transduction.

**Figure 9:**
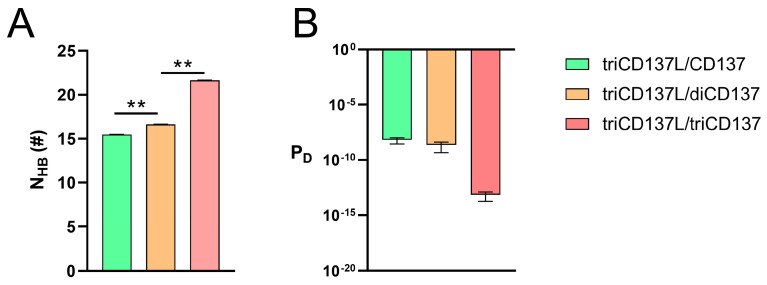
Stability of the complex increased as more CD137 bound to triCD137L. (**A**) N_HB_ and (**B**) P_D_ between one CD137L and the remaining complex parts. All data are shown as mean ± SEM (*n* ≥ 3), and ** indicates *p* < 0.001.

To investigate the effect of trimeric CD137L-induced CD137 trimerization on the stability of CD137L, we performed calculations to determine the N_HB_ and P_D_ between a single CD137L molecule and the remaining components of the complex. Of note, N_HB_ increased, whereas P_D_ decreased with increasing CD137 binding (Figure 9). These findings suggest that this process enhances the binding affinity of CD137L to CD137 but also contributes to the stability of the trimeric CD137L complex during the evolutionary process of CD137L-induced CD137 trimerization to form hexameric complexes.

## 3. Discussion

CD137 and its ligand CD137L play crucial roles in stimulating immune responses, with lack of CD137 resulting in immune dysregulation and increased risk of lymphomagenesis [43]. This interaction has attracted significant interest as a potential target for immunotherapy [44,45]; however, clinical trials involving agonistic antibodies targeting CD137 have largely failed because of systemic toxicity issues. Thus, gaining a comprehensive understanding of the relationship between CD137 and CD137L is essential for elucidating the signaling mechanisms within this critical receptor-ligand system and for developing agents that are both more effective and less toxic. In this study, we simulated three different states of CD137L (monomers, dimers, and trimers) to explore the physiological mechanisms underlying trimerization. Our investigation focused on the binding affinities of these states to CD137. We revealed that the evolution of the binding of the CD137L trimer to its monomeric counterpart can lead to the formation of a hexamer. Moreover, MD simulations indicated that the A’B’ loop of CD137L plays a significant role in its trimerization, but also in its interaction with CD137. Although the binding affinity of the CD137L trimer to CD137 was slightly lower than that of the dimer form, the trimer exhibited higher thermal stability. Furthermore, in the evolutionary process of CD137L-induced CD137 trimerization to form hexameric complexes, an increasing number of bound CD137 molecules enhanced the binding affinity and stability of the CD137L/CD137 interaction and the structure of trimeric CD137L.

For ligands belonging to the TNF family, the trimer stability and oligomerization status are key factors that influence receptor activation [46]. For instance, the small-molecule BIO8898 disrupts the TNF family cytokine CD40 ligand protein-protein interface by intercalating deeply between the two subunits of the CD40L homotrimer, thus inhibiting receptor binding [34]. Additionally, a well-structured aggregate formed by the small-molecule inhibitor JNJ525 induces a change in the quaternary structure of TNF-α, which in turn disrupts the interaction between TNF-α and its receptors [47]. To date, no small-molecule inhibitors that can disrupt the trimeric structure of CD137L have been developed, representing a promising new field of research.

Gilbreth et al. suggested that CD137L expresses a biologically active state in a stable trimeric form [37]. However, the concentration of CD137L in the solution used for testing was 2.3 mg/mL, which is far beyond the rational physiological concentration that blocks the reversible conversion of trimers to monomers. Interestingly, studies have indicated that (soluble) TNF is unstable at physiological concentrations (pg/mL–ng/mL) and gradually converts into inactive forms, such as monomeric TNF, in both buffered solutions and serum [35]. The monomeric and dimeric forms of CD137L were confirmed in physiologically expressed CD137L, with the extracellular cysteine residue (Cys51) forming a disulfide bond between monomers, leading to dimerization [48]. Thus, the dissociation of CD137 trimers results in dimers and monomers at physiological concentrations, and the denatured dimer and monomer CD137L forms can refold into an active trimeric protein state.

Our results showed that the trimer CD137L possesses higher thermal stability than the dimer, as shown by the enhanced interaction between the two monomers with increased H-bonds and buried SASA. Moreover, Chin et al. reported a high degree of flexibility of the A’B’ loop of triCD137L in the absence of a binding partner, which agreed with our findings that the flexibility of this loop is significantly impacted by the extensive movement of the B’ strand [30]. Indeed, the flexibility of the A’B’ loop was increased in the dimer form, which resulted in weaker stability. The H-bonds formed by LEU203 with GLN94, ARG202 with ASP112 or GLY231, and ARG221 with GLU191 are important for trimerization and are responsible for the higher thermal stability of triCD137L. Won et al. found that Q89A, L115A, H205A, V240A, P242K, P242E, and P242D mutations had no significant effects on CD137L trimerization [49]. Although the occupancy of the H-bonds between HSD205 and GLY114 increased from 14% in the dimer to 26% in the trimer, the relatively low occupancy indicated lower importance. These experimental data support our results, as these residues do not play an important role in trimerization, as revealed by FMD.

CD137 is composed of four domains: CRD1, CRD2, CRD3, and CRD4. Although CRD1 is not essential for CD137L binding, CRD2 and CRD3 serve as primary binding sites [50]. Previous studies have indicated that homotrimeric CD137L exhibits a significantly higher affinity for CD137 (1.2 nM) than its monomeric form (55.2 nM), highlighting the potential relevance of ligand conformation in modulating receptor interactions [48]. We revealed that the binding affinity of triCD137L was lower than that of diCD137L, but higher than that of the monomeric form. These results indicate that both dimerization and trimerization enhance the binding affinity of CD137L for CD137. We found that the conformational change in the A’B’ loop plays a key role in regulating this interaction. In the trimer configuration, the A’B’ loop of the primary CD137L is situated closer to the adjacent minor CD137L. Conversely, the dimer is positioned closer to the receptor. Although the binding affinity of the trimer to CD137 is weaker than that of the dimer, both monomers of the trimer are more tightly bound to each other, as characterized by the small angle γ and high number of H-bonds, which is benefit for structure stability. Our findings suggest that steric hindrance is a key factor in the reduced binding affinity of trimeric CD137L. The increased proximity between primary and minor CD137L monomers limits the flexibility of the A’B’ loop, which is essential for receptor engagement. Experimental and structural evidence also highlights the critical role of the A′B′ loop in CD137/CD137L interactions. The A′B′ loop exhibits flexibility in the unbound state but stabilizes upon receptor binding, with key residues like GLY114 and LEU115 directly involved in binding [30,35,36]. Mutation studies, such as L115G, further demonstrate the functional importance of this region by significantly reducing binding affinity [49]. These findings collectively underscore the essential regulatory role of the A’B’ loop in CD137L trimer stability and receptor binding affinity. Because of the large difference in the binding area between CD137 and the primary or minor CD137Ls, whether the minor CD137L is important for the binding of CD137 is controversial [37]. We found only one or two H-bonds between the minor CD137L and CD137, which were intermittent. The minor CD137L contributes to the binding of CD137L to CD137 but is of limited support. However, we observed that diCD137L and triCD137L exhibit a significantly higher affinity for CD137 compared to monoCD137L, and triCD137L exhibits a significantly higher affinity for CD137 than its monomeric form, as verified by surface plasmon resonance analyses [48]. This enhanced affinity is likely due to CD137L dimerization and trimerization, which stabilizes the conformation of CD137L and subsequently reinforces the stability of the CD137L-CD137 binding interface.

Cross-linking of TNF receptors (TNFRs) through trimeric ligands is generally recognized as the primary mechanism for initiating receptor activation [46,51]. When CD137 is activated by the binding of CD137L trimers or cross-linking with agonist monoclonal antibodies, TRAF1–3s are quickly recruited to the cytoplasmic domain of CD137. This recruitment likely occurs as homo- and/or heterotrimeric complexes with various configurations that initiate the formation of CD137 signalosomes [39]. The TNF-α homotrimer is mixed with its receptor TNFR1 over a range of ratios (1:1.2, 1:2.2, and 1:3.2), resulting in TNF-α homotrimer bound to one, two, and three TNFR1s, respectively [34]. These results suggest that the amount of TNFR1 bound to the TNF-α trimer depends on the relative concentration of TNFR1. Because CD137 exists primarily as a monomer, CD137 is bound individually to the triCD137L, eventually forming a hexamer. In analogy to TNF-α/TNFR1, the formation of the hexamer (a triCD137L bound to three CD137s) includes two intermediate forms: triCD137L bound to either one or two CD137s; however, this evolution has not been observed for CD137L/CD137. Nonetheless, we revealed that the binding affinity of CD137L to CD137 gradually increases in evolution from triCD137L binding to a single CD137 molecule, to two CD137 molecules, and eventually to a hexamer. The results of the number of H-bonds and P_D_ between one CD137L and the rest of the complex further indicated that the stability of the complex increases as more CD137/triCD137L are bound. This evolutionary transition in complex formation plays a crucial role in determining signaling specificity and pathway activation in a physiological context. The assembly of the triCD137L/triCD137 hexamer enhances receptor clustering, which in turn promotes the formation of the CD137 signalosome [39].

While our simulations provide key insights into trimer stability and receptor-ligand interactions, we acknowledge that short-timescale molecular dynamics (MD) simulations may not fully capture rare conformational transitions or long-lived intermediate states. To address these limitations, enhanced sampling techniques, such as those employed in SEEKR2 and other milestoning-based methods, could be particularly useful. SEEKR2 leverages multiscale milestoning to accelerate sampling while maintaining accuracy in predicting ligand binding/unbinding kinetics, which has been effectively applied to HSP90, JAK-STAT inhibitors, and threonine-tyrosine kinase [52,53,54,55]. Applying SEEKR2 to our system could provide a more comprehensive kinetic analysis of CD137L/CD137 binding and stability, identifying rare conformational events that may be missed in standard FMD simulations. Additionally, weighted ensemble simulations and Markovian milestoning with Voronoi tessellations have demonstrated effectiveness in ranking ligand binding kinetics, making them promising approaches for studying long-lived states and binding free energy landscapes in the CD137L/CD137 interaction [56,57,58]. These methods could allow for more accurate predictions of residence times and binding kinetics, which are crucial for rational drug design targeting CD137. DeepWEST, a deep-learning-assisted weighted ensemble simulation framework, has demonstrated success in capturing long-lived states and complex protein dynamics [59]. Applying DeepWEST to CD137L trimerization could reveal rare but functionally important conformational changes that influence receptor engagement. This method would complement traditional MD simulations by improving sampling efficiency and identifying key structural transitions that regulate CD137 activation.

Molecular dynamics (MD) simulations excel at capturing subtle structural changes that can have profound functional consequences, which traditional experimental techniques often miss [60]. For example, MD simulations have revealed how the D614G mutation in the SARS-CoV-2 spike protein disrupts a single hydrogen bond, increasing receptor flexibility and enhancing viral infectivity [61]. Similarly, they have shown that minor conformational shifts in the hyaluronan-binding domain of CD44 significantly boost ligand affinity [62]. In studying CD137L oligomeric states, MD simulations provide detailed molecular interaction trajectories, making them ideal for assessing binding affinity and stability. However, experimental validation remains essential. Techniques such as Förster resonance energy transfer (FRET), fluorescence-assisted high-performance size-exclusion chromatography (HP-SEC), and mutational analyses could confirm the binding dynamics and stability of different CD137L states. Our findings emphasize the importance of controlling oligomeric states to optimize CD137 signaling, with direct implications for next-generation agonists. Future designs could mimic dimeric or trimeric CD137L to enhance receptor engagement while incorporating tumor-microenvironment-sensitive linkers or controlled oligomerization strategies to improve specificity and minimize systemic toxicity. Targeting the A’B’ loop may further enhance receptor binding, while tumor-specific linkers (pH- or protease-sensitive) could regulate oligomerization to prevent off-target activation. Additionally, our study provides a foundation for developing CD137 agonists that function independently of FcγRIIb, overcoming a major limitation of current therapies. By integrating these strategies, future CD137-based immunotherapies could achieve greater efficacy with reduced toxicity, expanding their clinical applications.

In summary, our study elucidates the mechanism of CD137L trimerization and the structural basis of CD137L binding to CD137, highlighting the evolutionary transition to hexamer formation. The present work provides valuable insights for the design of small-molecule drugs aimed at destabilizing the trimeric CD137L structure or acting as CD137 agonists, providing new foundations for cancer immunotherapy and the treatment of autoimmune diseases.

## 4. Materials and Methods

### 4.1. System Setup

The crystal structure of the human triCD137L was obtained from the Protein Data Bank (PDB ID: 6MGE). Missed residues on the monomer CD137Ls were added using the Schrödinger protein preparation wizard in Maestro 11.5 [63]. The triCD137L included three CD137L monomers (chains A, B, and C). We built three diCD137L complexes by removing one monomer CD137L from the triCD137L using Visual Molecular Dynamics (VMD) 1.9.3 [64], resulting in diCD137L-1, diCD137L-2, and diCD137L-3, which were composed of chains A and B, chains A and C, and chains B and C, respectively (Figure 1A and Supplementary Table S1).

The crystal structure of human triCD137L bound to three CD137L molecules was obtained from the Protein Data Bank (PDB ID: 6A3V). As this crystal structure contained four repeats of the CD137L/CD137 hexamer, we only selected a hexamer consisting of chains A, B, C, D, E, and F. In this crystal structure, chains A, C, and E represented the three monCD137Ls and chains B, C, and D represented the three CD137s. To align with the above complex, we renamed these chains: chain E was renamed B and chain B was renamed E. In the hexamers, CD137 were bound to two monCD137Ls. However, CD137 did not bound uniformly to both monCD137Ls. We named CD137L, which bound mainly to CD137, primary monCD137L, and CD137L with a smaller binding surface to CD137, minor monCD137L. The next system was constructed using VMD 1.9.3. First, we selected one CD137 of the three and bound the primary monCD137L, thus building three CD137L/CD137 complexes. Next, we added another CD137L bound to CD137, a minor monCD137L, to the CD137L/CD137 complex and thus set up three complexes of the dimer CD137L bound to CD137 and diCD137L/CD137. We then added the last CD137L to diCD137L/CD137 to form three complexes of the trimer CD137L bound to CD137 and triCD137L/CD137. CD137L did not bind to CD137 and thus we named it free CD137L. To simulate the process of trimer CD137L binding to one, two, and three CD137s, and finally forming a hexamer, we added a second CD137 to the triCD137L/CD137 system, resulting in three complexes of triCD137L bound to two CD137s (triCD137L/diCD137). Finally, we named the hexamer in which triCD137L bound to three CD137s (triCD137L/triCD137) (Figure 1B and Supplementary Table S1).

To simulate the continuation of the protein chain, terminal patches of ACE and CT3 were incorporated at the N- and C-terminal ends of CD137L and CD137 proteins, respectively. These 17 complexes were solvated in TIP3P water using VMD, ensuring a minimum distance of 15 Å from any protein atom to the walls. Subsequently, the systems were neutralized with 150 mM NaCl to replicate the physiological environment.

### 4.2. MD Simulations

MD simulations were conducted using NAMD 2.14, employing all-atom MD with periodic boundary conditions, a 2 fs time step, and the CHARMM27 force field, which included CMAP correction for the backbone [65]. The particle mesh Ewald method was used for electrostatic interactions, with a cutoff of 12 Å for both electrostatic and van der Waals interactions. The systems underwent energy minimization with the protein held fixed for the first 15,000 steps, followed by another 15,000 steps with the backbone fixed before allowing all atoms to move freely for the final 15,000 steps. These energy-minimized systems were gradually heated from 0 to 310 K over 0.1 ns and equilibrated for 40 ns under controlled pressure and temperature conditions. The temperature was maintained at 310 K using Langevin dynamics and the pressure was maintained at 1 atm using the Langevin piston method. To evaluate the thermostability of the complexes, free MD simulations were conducted [66]. These free MD simulations were performed in a microcanonical ensemble, indicating that the temperature and pressure were not maintained at fixed values. Free MD simulations provide insights into the system’s inherent flexibility and thermal stability that are not easily accessible through conventional equilibrium MD simulations [66]. The equilibrated structure served as the starting conformation for FMD simulations, which lasted for 100 ns.

### 4.3. Data Analysis

Data from the resulting trajectory files of the MD simulations were analyzed using the VMD tools. We calculated the Cα RMSD and SASA (with a 1.4 Å probe radius) to assess conformational changes and hydrophobic core exposure, respectively. A H-bond was counted when the donor-acceptor distance was less than 3.5 Å and the donor-hydrogen-acceptor angle was below 30°. The occupancy of H-bonds was determined as the ratio of the bond survival time to the simulation period. We also used P_D_, as outlined in our previous studies, to evaluate binding stability [67]. The Molecular Mechanics Poisson–Boltzmann Surface Area Method was employed to calculate the binding energy during FMD simulations [68].

### 4.4. Statistical Analysis

Data were analyzed using the unpaired Student’s *t* test or one-way analysis of variance with a multiple comparison test. Values were considered statistically significant at *p* < 0.05.

## Data Availability

The crystal structures used in this study are available from the Protein Data Bank (PDB) with the following IDs: 6MGE and 6A3V (https://www.rcsb.org/structure/6MGE; https://www.rcsb.org/structure/6A3V (accessed on 12 June 2022)). The molecular dynamics simulations were performed using VMD (version 1.9.3) for visualization, available at https://www.ks.uiuc.edu/Research/vmd/ (accessed on 6 June 2022), and NAMD (version 2.14) for the simulations, available at https://www.ks.uiuc.edu/Research/namd/ (accessed on 6 June 2022). PyMOL (version 2.6.0) was used for additional structural analysis and can be accessed at https://pymol.org/2/ (accessed on 18 December 2022). The CHARMM27 force field used in the simulations was obtained from the official CHARMM website, https://www.charmm.org/ (accessed on 6 June 2022).

## Supplementary Materials

The following supporting information can be downloaded at: https://www.mdpi.com/article/10.3390/ijms26051903/s1.
