# Supplementary material for: MD Simulation Reveals a Trimerization-Enhanced Interaction of CD137L with CD137
Source: Int J Mol Sci. 2025 Feb 22;26(5):1903. doi: 10.3390/ijms26051903 (PMC11899465; doi:10.3390/ijms26051903)
Supplement: Supplementary file 1 [file ijms-26-01903-s001.zip › ijms-3409739-supplementary.pdf]

Supplementary Materials

MD Simulation Reveals a Trimerization-Enhanced Interaction of CD137L with CD137

Hefeng Wang, Jianhua Wu, Ying Fang * and Quhuan Li *

School of Biology and Biological Engineering, South China University of Technology, Guangzhou 510006, China; 201910108090@mail.scut.edu.cn (H.W.); wujianhua@scut.edu.cn (J.W.)
* Correspondence: yfang@scut.edu.cn (Y.F.); liqh@scut.edu.cn (Q.L.)

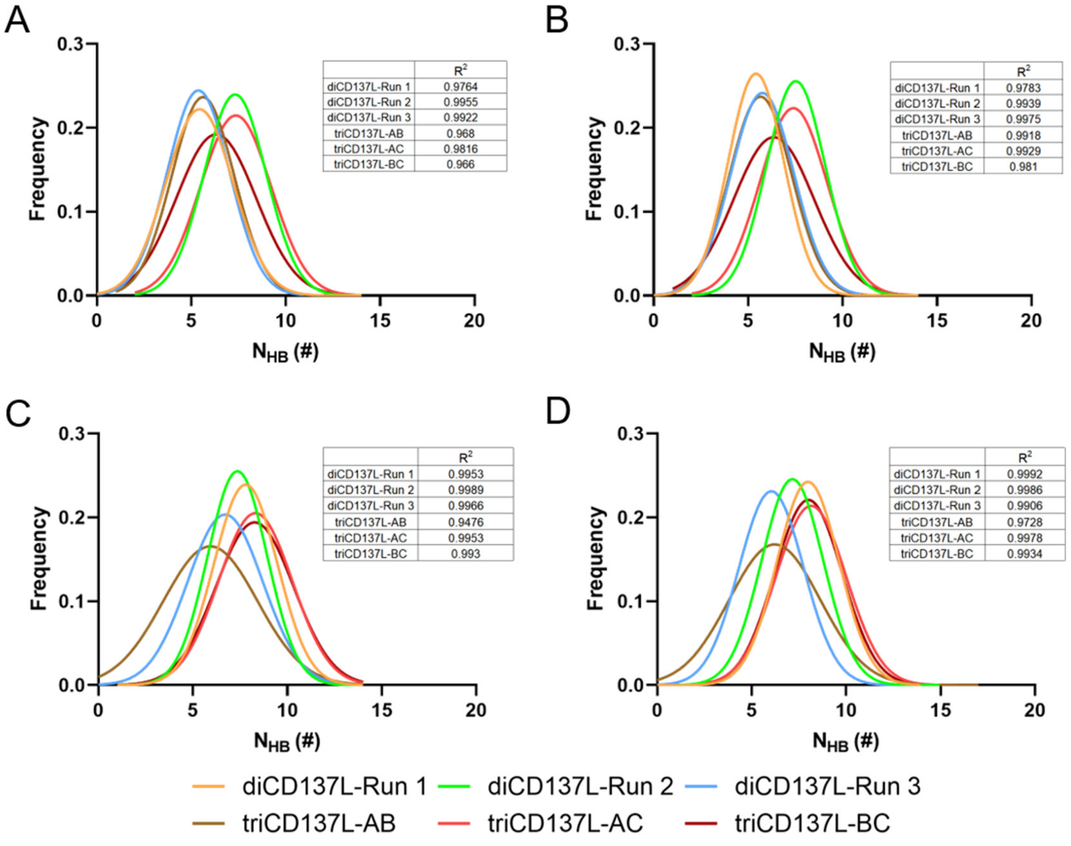

Figure S1. Distribution of N_{HB} during equilibrium and free MD simulations. (A) The frequency distribution of the number of hydrogen bonds during the first 20 ns of the 40 ns equilibrium process. (B) The frequency distribution of the number of hydrogen bonds throughout the entire 40 ns equilibrium process. (C) The frequency distribution of the number of hydrogen bonds during the first 50 ns of the 100 ns free MD simulations. (D) The frequency distribution of the number of hydrogen bonds throughout the entire 100 ns free MD simulations. The R^2 values of the Gaussian fits are displayed.

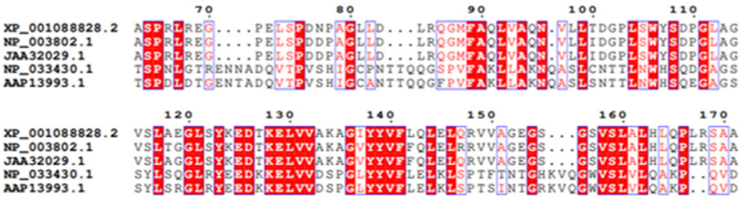

NCBI RefSeq	Species
XP_001088828.2	Macaca mulatta
NP_003802.1	Homo sapiens
JAA32029.1	Pan troglodytes
NP_033430.1	Mus musculus
AAP13993.1	Rattus norvegicus

Figure S2. Conservation analysis of A′B′ and B′B′ loops in CD137L across species.

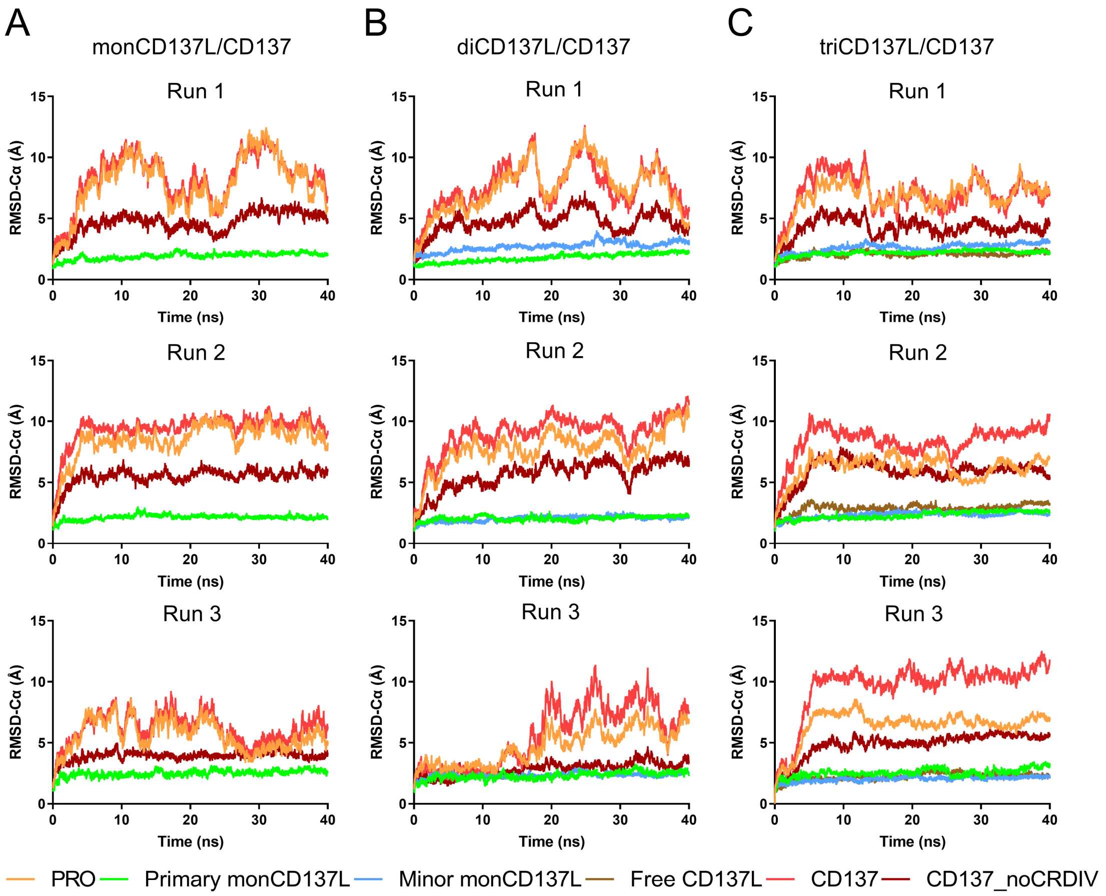

Figure S3. Time course of Cα RMSD of different CD137L/CD137 complexes. (**A**) MoCD137L/CD137, (**B**) diCD137L/CD137, and (**C**) triCD137L/CD137 complex systems without the CRD4 domain of CD137. Three complexes of each system went through 40 ns equilibrium. Orange: protein (PRO); green: CD137L that bound primarily to CD137 (Primary monCD137L); blue: CD137L that had minor binding surface with CD137 (Minor monCD137L); brown: CD137L that did not bound with CD137 (Free CD137L); red: CD137; dark red: CD137 without CRD4 domain (CD137_noCRDIV). The fluctuations observed in these graphs unequivocally indicate that CRD4 is the primary driver. With the exception of CRD4, the remaining components of the complex exhibit remarkable stability.

Table S1: Setting-up of simulation system

Research design	Results and figures	Receptor	Ligand*	Simulation System
**Step 1. Simulation of dimeric and trimeric CD137L** **Non Receptor** **Dimeric/Trimeric Ligand**	Result 1,2 Figure 2,3	Non	diCD137L-AB	diCD137L-Run 1
			diCD137L-AC	diCD137L-Run 2
			diCD137L-BC	diCD137L-Run 3
			triCD137L-ABC	triCD137L-Run 1,2,3
**Step 2. Simulation of the binding of a CD137 to CD137L in different states of aggregation** **Monomeric Receptor** **Monomeric/Dimeric/Trimeric Ligand**	Result 3,4 Figure S1,4,5,6	CD137	monCD137L-A	monCD137L/CD137-Run 1
			monCD137L-B	monCD137L/CD137-Run 2
			monCD137L-C	monCD137L/CD137-Run 3
			diCD137L-AB	diCD137L/CD137-Run 1
			diCD137L-AC	diCD137L/CD137-Run 2
			diCD137L-BC	diCD137L/CD137-Run 3
			triCD137L-ABC	triCD137L/CD137-Run 1
			triCD137L-ABC	triCD137L/CD137-Run 2
			triCD137L-ABC	triCD137L/CD137-Run 3
**Step 3. Simulation of trimeric CD137L binding to different amounts of CD137** **Monomeric/Dimeric/Trimeric Receptor** **Trimeric Ligand**	Result 5 Figure 7,8,9	CD137	triCD137L-ABC	triCD137L/CD137
		diCD137	triCD137L-ABC	triCD137L/diCD137
		triCD137	triCD137L-ABC	triCD137L/triCD137

Notes: * A, B, and C refer to chain A, chain B, and chain C in CD137L.
